# Post-traumatic Distress in Adults with Congenital Heart Disease: Protective Factors and Clinical Implications

**DOI:** 10.1007/s12529-024-10332-z

**Published:** 2024-11-12

**Authors:** Annika Freiberger, Caroline Andonian-Dierks, Jürgen Beckmann, Sebastian Freilinger, Peter Ewert, Peter Henningsen, Harald Kaemmerer, Niko Kohls, Cristina Richter, Maximilian Huber

**Affiliations:** 1 Department of Congenital Heart Disease and Pediatric Cardiology, German Heart Center Munich, TUM University Hospital, Munich, Germany; 2 Department of Psychosomatic Medicine and Psychotherapy, School of Medicine, Technical University Munich, Munich, Germany; 3 Department of Sport and Health Sciences, Technical University Munich, Munich, Germany; 4 School of Human Movement and Nutrition Sciences, University of Queensland, Brisbane, QLD Australia; 5 Department of Sport and Health Sciences, Chair of Preventive Pediatrics, Technical University of Munich, Munich, Germany; 6 Division of Health Promotion, Faculty of Applied Natural Sciences and Health, Coburg University of Applied Sciences, Coburg, Germany

**Keywords:** Adults with congenital heart disease, Post-traumatic stress disorder, Resilience, Social support, Sense of coherence

## Abstract

**Background:**

Due to various reasons explored in previous studies, adults with congenital heart disease (ACHD) are at risk of developing post-traumatic stress symptoms (PTSS). The aim of this study is to explore multiple potential psychosocial protective factors in ACHD and to understand their role in different complexities of congenital heart disease (CHD) and PTSS.

**Method:**

This study was part of the “ABS-AHF” study, where 234 ACHD were recruited from November 2021 to August 2022 at a tertiary CHD care center. Data were collected on PTSS (PDS), resilience (RS-13), sense of coherence (SOC-L9), and social support (F-SozU K-14).

**Results:**

The mean scores were 70.55 + / − 12.31 [21–91] for resilience, 35.83 + / − 4.81 [15–60] for sense of coherence (SOC), and 4.30 + / − 0.79 [0.93–5] for social support. SOC (OR, .91; *p* = .024 [.84; 9.98]) and social support (OR, .48; *p* = .001 [.29; 7.96]) were shown to reduce the likelihood of PTSS. Low resilience (OR, 2.40, *p* = .0248 [1.18; 5.18]) seems to increase this likelihood.

**Conclusion:**

Integrating parents and relatives early on seems to be an important protective resource. Parental support in childhood affects the development of SOC which is in line with social support related to lower PTSS. With regard to resilience and SOC, a brief and manageable screening option for personal resources is available to refer potentially vulnerable patients to specialized psychosocial care. Care offers should address coping styles and life with CHD. Offering multidisciplinary care, integrating the patient’s social network, and education for patients to increase resilience and SOC might provide a way to enhance psychosocial outcomes, quality of life, and adherence in ACHD.

## Introduction

Congenital heart disease (CHD) is the most common birth defect worldwide. Throughout the last decades, substantial advances in medical treatment and cardiac care led to a growing number of patients with CHD reaching adulthood with more than 97% in developed countries [1]. Due to the chronic condition in many cases, adults with CHD (ACHD) have to cope with repeated check-ups, hospitalization, and medical interventions, potentially leading to permanent fear, stress, and uncertainty [2, 3].

Besides being at high risk for developing depression and anxiety [4], current research shows that ACHD present an elevated prevalence of post-traumatic stress symptoms (PTSS) compared to the general population (about 3.5% of post-traumatic stress disorder (PTSD)) [5, 6] with a prevalence rate of 20.5% [7]. PTSS can arise after cardiac-related events, such as surgery or symptoms like arrhythmia, and may include flashbacks, avoidant behavior, sleep problems, and emotional numbing but do not represent a manifest diagnosis of PTSD [8]. However, addressing PTSS is crucial as it can significantly impact the course of CHD [9–11]. Previous research has shown a connection between the underlying clinical state and the risk of developing PTSS, though the exact reasons why some patients develop PTSS remain unclear [7]. Investigating protective and risk factors, such as sense of coherence (SOC), resilience, and social support, is essential for preserving or restoring health [12–14].

SOC is a central element in Antonovsky’s salutogenetic theory and reflects individuals’ conviction that their lives are understandable, manageable, and meaningful [15]. Resilience can be defined as a process and outcome of a successful adaptation to different challenging experiences in life [8] or the ability to “bounce back” from adversity [16]. Resilience has been shown to be potentially important when living with chronic conditions or when recovering from illness [17].

Both SOC and resilience have been found to be of protective character regarding the development of PTSS [14, 18]. Perceived social support contributes to prevention and treatment of cardiac diseases [19]. A strengthening of social support was found to contribute to improving or restoring the SOC for people with mental health problems [20]. Specifically, social support has been shown to decrease the risk of experiencing PTSS [13, 21–23]. Social support consists of accessible support through other individuals, groups, or community and a network of people helping in case it is needed [13].

Considering the adversities faced by ACHD early in life, such as repeated surgeries and hospitalizations, it becomes crucial to target these protective factors early on for the prevention of PTSS. However, research on the development and prevention of PTSS in ACHD is limited. Therefore, this study aims to explore potential protective factors, such as social support, SOC, and resilience, in ACHD and understand their role in relation to the complexity and severity of CHD and PTSS in this patient population. Identifying protective factors in PTSS can help identify individuals at risk, predict prognostic factors, and promote bio-psycho-social health and psychological endurance and resilience to prevent PTSS in ACHD.

## Material and Methods

The present cross-sectional, single-center study was carried out at a large-volume tertiary care center, which specializes in all different forms of CHD. It is part of the ongoing research project “ABS-AHF” conducted by the working group of psychocardiology at the German Heart Center Munich [7]. Participants were enrolled between November 2021 and August 2022 at the Department of Congenital Heart Disease and Pediatric Cardiology. Inclusion criteria were (I) confirmed diagnosis of CHD, (II) participant aged 18 years and older, (III) sufficient physical, cognitive and language capabilities to complete self-report questionnaires, and (IV) German speaking. Reasons for exclusion were severely impaired cognitive abilities.

The study package consisted of (I) study information, (II) informed consent, and (III) questionnaires. Information related to medical parameters was extracted from medical records. Informed consent was obtained from each patient, and procedures were performed in accordance with the Declaration of Helsinki. The study has been approved by the Ethical Committee of the Technical University of Munich in September 2021 (570/21 S). The present study was carried out according to the STROBE guidelines [24].

A self-devised questionnaire obtained sociodemographic information. As ACHD is a very specific and diverse field, it was not possible to use a standardized and validated questionnaire. For this purpose, a questionnaire was specifically devised in cooperation with the Chair of Behavioral Epidemiology at the Technical University of Dresden and the Department of Congenital Heart Disease and Pediatric Cardiology, German Heart Center Munich. This questionnaire included items related to the individuals’ sociodemographic status, underlying CHD, treatment status, comorbidities, and AHA-classification. This questionnaire has been used routinely at the German Heart Center for more than 10 years and serves our clinical projects and disease registries. As patient-reported questionnaires do not provide a clinical diagnosis of PTSD, the results of the assessment are referred to as subjectively reported PTSS. For the assessment of PTSS, the Post-traumatic Diagnostic Scale (PDS) [25] was used. Patients were asked to respond referring to their worst experience related to their CHD. The PDS is a self-report measure of PTSS based on diagnostic criteria of the fifth edition of the Diagnostic and Statistical Manual of Mental Disorders (DSM-5) [26]. It consists of 17 questions based on a 5-point Likert-type scale from 0 (not at all) to 4 (severe) to assess the symptoms the patient is suffering from. It includes each of the 20 DSM-5 PTSD symptoms and inquires their onset and duration. A higher score indicates a higher severity of PTSS, where a clinically relevant cut-off value of 36 resulted in high sensitivity and specificity [27]. A cut-off value of 11 is used as an indicator for a potential diagnosis of PTSS. The German version of the PDS is a valid and reliable measure of PTSS differentiating both symptom severity and diagnostic acuity [27].

For the assessment of resilience, the RS-13 was used. The RS-13 is the short version of the RS-25 questionnaire which has been developed by Wagnild and Young [28]. The questionnaire persists of a two-dimensional structure referring to the two subfacets “personal competence” and “acceptance of self and life.” The RS-13 consists of 13 items on a 7-point scale (1 = I do not agree, 7 = I totally agree with different statements) and has been validated in representative samples [29]. The final score can be grouped into low (less than 67 points), moderate (67–72 points), or high resilience (73–91 points) [29].

SOC was assessed with the SOC-L9. The SOC-L9 is the short-form scale consisting of nine items derived from the original 29-item questionnaire (SOC-29). Responses are rated on a 7-point Likert scale and can be summed [30]. The SOC-L9 is a highly validated measure that shows good psychometric values [31].

Perceived social support was assessed with the 14-item short version of the F-SozU, which is a standard instrument for assessing social support in German speaking populations. Items are rated on a 5-point Likert scale (1 = does not apply at all to 5 = totally applies). To calculate the final value, all items are summed up and divided by the number of items with higher values indicating higher social support. The F-SozU K-14 has been validated and has shown very good psychometric properties [32]. Additionally, perceived parental support during childhood was assessed with a scale ranging from 1 (very little support) to 10 (highly supported) on the self-devised questionnaire.

Medical information was obtained from various medical records. To consider the severity of the heart defect and its patho-physiological consequences, patients were classified according to the ACHD AP Classification (CHD Anatomy + Physiological Stage = ACHD AP Classification) introduced by Stout et al. [33]. The patho-anatomic classification consists of groups I to III (simple, moderate, and complex/great complexity). The patho-physiology or disease severity classification consists of four groups: A, B, C, and D. This classification is based on several medical functional parameters, such as functional class and ventricular or valvular function. Patients who did not fit into any of these classifications were summarized into “other,” such as patients with Marfan syndrome, and were not considered in statistical analysis. Other biomedical parameters, such as cyanosis status, Fontan circulation, or arrhythmia, were also assessed and have been previously published [7].

### Statistics

All calculations were performed using SPSS Statistics 28.0 (IBM Inc., Armonk, NY, USA). Descriptive analysis was used to calculate measurements of central tendency and distribution. For the assessment of associations between medical parameters and protective factors or PTSS, Spearman’s rank correlation or Pearson’s correlation was used respectively. Logistic regression models, odds ratios (OR), and 95% confidence intervals (95% CI) were used for a better understanding of medical and mental associations. To test potential moderation effects of the protective factors, different moderation models were used in accordance with procedures described by Hayes and the PROCESS macro for SPSS. Moderation effects were assessed by evaluation of 95% CI produced by bootstrapping (bootstrapping = 5000). As only patient-reported data was used in this study, missing data was reported and excluded in analysis to not distort the patients’ reality. Data was considered as missing if the questionnaire was not filled out at all. All occurring *p* values and tests for significance were performed two-sided. Statistical significance was indicated by a *p* value < 0.05.

## Results

### Participant Characteristics

A total of 569 study packages were distributed at the Department of Congenital Heart Disease and Pediatric Cardiology, and 264 questionnaires were returned (response rate, 46%). Due to missing consent, 30 questionnaires had to be excluded. For the final statistical assessment, 234 participants were included. Sociodemographic characteristics are presented in Table 1. Clinical characteristics are shown in Table 2.

**Table 1:** Sociodemographic variables

Variables	Value, number (%)
Age, years	35.2 ± 10.8 [18–66]
Gender	
Female	109 (46.6)
Male	124 (53.0)
Missing	1 (0.4)
Marital status	
Married	97 (41.5)
Divorced	3 (1.3)
Engaged	56 (23.9)
Single	74 (31.6)
Widowed	1 (0.4)
Missing	3 (1.3)
Level of school education	
No schooling completed	1 (0.4)
Lower secondary school degree	33 (14.1)
Secondary school degree	56 (23.9)
University entrance qualification	44 (18.8)
General higher education entrance qualification	97 (41.5)
Missing	3 (1.3)
Financial standing (self-assessed)	
Good or very good	156 (66.7)
Neither good or bad	63 (26.9)
Bad or very bad	11 (4.7)
Missing	4 (1.7)
Children	
Yes	77 (32.9)
No	155 (66.2)
Missing	2 (0.9)
Employment status	
Fulltime employed	152 (65.0)
Part-time employed	52 (22.2)
Not working	15 (6.4)
Unemployed	2 (0.9)
Retired	4 (1.7)
Missing	9 (3.8)

**Table 2:** Clinical characteristics and clinical classification according to Stout et al. [33]

Variables	Values, number (%)
Severity of CHD according to patho-anatomy I. Simple II. Moderate III. Complex/great complexity Other	30 (12.8) 107 (45.7) 74 (31.6) 23 (9.9)
Severity of CHD according to patho-physiology A B C D Other	26 (11.1) 115 (49.1) 70 (29.9) 11 (4.7) 12 (5.1)

### Mental Health Status and Protective Factors

Patients were requested to respond to the questions in the PDS only if they have experienced any traumatic event related to their CHD, while the remaining participants were considered as not exhibiting or not willing to address PTSS. Out of 234 participants, 173 completed the PDS. Of all patients, 20.5% (*n* = 48) presented scores higher than 11, potentially indicating the presence of PTSS, while 6.8% (*n* = 16) were above the cut-off value for moderate to severe PTSS. The F-SozU K-14 was completed by 222 participants. The mean score for social support (F-SozU K-14) was 4.3 + / − 0.79 [0.93–5.0]. For SOC (SOC-L9), 213 participants completed the questionnaire, and the mean score was 35.82 + / − 4.81 [15–60]. For resilience (RS-13), the mean score was 70.55 + / − 12.31 [21–91]. The RS-13 score can be categorized into low, medium, and high resilience. In total, 214 participants completed the RS-13. Thereof, 31.8% (*n* = 68) can be classified as low, 17.8% (*n* = 38) as medium, and 50.5% (*n* = 108) as highly resilient according to the cut-off values.

### Associations Between Medical and Psychological Parameters

Bivariate correlations among the study variables are presented in Table 3. Age and gender were not correlated with any of the main study variables. Social support, resilience, and SOC were inversely correlated with PTSS severity. Additionally, social support and SOC were positively correlated with patho-physiology classification. Social support and SOC were higher in more severe functional classes. For PTSS severity, a negative correlation with this classification was found. PTSS severity was lower in more severe patho-physiology classes. Resilience was inversely correlated with patho-anatomical classification. More severe patho-anatomy classifications were associated with lower resilience. Social support during childhood was positively correlated with SOC. The more social support was experienced as a child, the higher were the SOC scores. Furthermore, current social support (F-SozU) as well as resilience was negatively correlated with PTSS severity.

**Table 3:** Bivariate Pearson’s correlations

	1	2	3	4	5	6	7
1. PTSS severity							
2. Resilience	− .182*^ƚ^						
3. Social support	− .261**^ƚ^	373**^ƚ^					
4. Sense of coherence	− .197*^ƚ^	.248**^ƚ^	.175*^ƚ^				
5. Age	− .112^ƚ^	.067^ƚ^	− .007^ƚ^	.030^ƚ^			
6. Social support during childhood	− .294**^ƚ^	.184**^ƚ^	.223**^ƚ^	.124*^ƚ^	− .089^ƚ^		
7. Gender	.133^‡^	− .016^‡^	.060^‡^	− .015^‡^			
8. Patho-physiology classification	.181*^‡^	− .133^‡^	− .110^‡^	− .005^‡^		− .033^‡^	
9. Patho-anatomy classification	.111^‡^	− .162*^‡^	− .061^‡^	.038^‡^		− .008^‡^	.172*^‡^

^*^p < .05; **p < .01. *PTSS*, post-traumatic stress symptoms, ^ƚ^bivariate Pearson’s correlation, ^‡^Spearman’s rank correlation

Looking at between-group differences according to patho-physiology classification, significant differences in PTSS severity were revealed (*p* = 0.018). A post hoc Bonferroni test showed differences between low and high functional severity of CHD: groups A versus D (*p* = 0.029) as well as B versus D (*p* = 0.014). The group with most severe CHD (Group D) showed higher PTSS scores than the other groups. No significant differences between resilience, SOC, or social support and patho-physiology classification were found (*p* > 0.05). Additionally, there were no significant differences between patho-anatomy classification groups in resilience (*p* > 0.05).

The logistic regression model investigating associations between social support, SOC, and resilience and the likelihood of PTSS was overall significant (*χ*^2^ = 18.169; *p* < 0.001). Two of three protective factors reduce the likelihood of developing PTSS in ACHD: social support (OR, 0.484; *p* = 0.001 [0.294; 0.796]), and SOC (OR, 0.910; *p* = 0.024, [0.839; 0.988]). Resilience was not significantly associated with a higher likelihood of presence of PTSS (*p* = 0.784). Looking at ACHD with high resilience (*n* = 82) compared to ACHD with low resilience (*n* = 57), low resilience was significantly associated with a higher likelihood of presence of PTSS (OR, 2.40; *p* = 0.0248 [1.177; 5.1802]).

### Moderation Analysis of Protective Factors

Based on the associations between the main study variables, two different moderation models were postulated: the patho-physiology classification served as independent variable, PTSS severity as dependent variable, and the three protective factors as moderator, respectively. Using social support as moderator between patho-physiology class and PTSS severity resulted in a non-significant model (*p* = 0.281). Taking SOC as moderator variable resulted in an overall significant model (*p* = 0.0065) predicting 14.84% of the variance in the target variable PTSS severity. However, no significant moderation effect of SOC was found for the interaction between patho-physiology classification and PTSS severity (*p* = 0.173).

## Discussion

This is the first study investigating various potentially protective factors and their association with PTSS in ACHD. The study revealed several novel findings regarding protective factors regarding the development of PTSS. Perceived social support and SOC are strongly associated with PTSS. Based on the findings, it can be assumed that perceived social support and SOC could reduce the likelihood and low resilience could increase the likelihood of the development of PTSS after perceived traumatic events in ACHD.

Interestingly, ACHD show strong psychosocial resources in terms of social support and resilience. Perceived social support was higher in our study population than in the general German population (4.3 + / − 0.79 vs. 3.97 + / − 0.68) [34]. In general, perceived social support has been shown to be a strong protective factor against the development of PTSS in various study populations [12, 22, 23]. In the present study, perceived social support during childhood was negatively associated with PTSS severity. As most of ACHD had medical treatment or check-ups during childhood, this could refer to their social support at the time of surgery and hospital stay. Furthermore, perceived social support during childhood was associated with higher scores in SOC and resilience. This is in line with non-ACHD research showing that experiencing social support in early years promotes the development of SOC [35]. However, SOC was lower in ACHD compared to the general German population [30]. A potential explanation for this might be the setting of data collection, as patients were approached during waiting time and were potentially nervous or anxious and had therefore a more negative outlook on their life at this moment.

SOC was associated with a lower likelihood of developing PTSS in our study. This might be due to several reasons. First, SOC is linked to a better quality of life, which presents a protective impact on dealing with chronic stress [36]. Furthermore, a strong SOC can lead to mobilizing and applying resistance sources and lead to solution seeking and consequently to a better coping with stressors [15, 35]. Moons et al. hypothesize that growing up with a CHD can influence the development of SOC positively. This might be because living with a CHD enhances the three parts of SOC: comprehensibility, meaningfulness, and manageability [37].

No differences between different patho-physiological classifications or structural CHD severity and SOC were found. The development of SOC is likely more influenced by the chronic condition of the CHD and living with a chronic disease from childhood onwards. This might also apply to other chronic diseases like diabetes [37].

Resilience was high according to the cut-off values and comparable to the general population [29]. Low resilience has been shown to be a strong factor contributing to a higher likelihood of PTSS. This finding is supported by studies in other populations [18, 38]. Additionally, resilience was associated with anatomic severity of the CHD. Resilience can be interpreted as either trait or state resilience [39]. In general, resilience has recently been defined as a developmental process by many researchers [39].

Social support has been shown to be a strong contributor the development of resilience [40], and social support during childhood was significantly associated with anatomic CHD severity in this study. However, patients with more severe CHD show lower scores in resilience, but this association appears to be rather weak. A meta-analysis by Lee et al. found that resilience seems to play a role in the course of various mental problems, such as depression, anxiety, and PTSD. It can be seen as a dynamic process protecting the individual in stressful situations as well as increasing therapeutic outcomes [39].

In general, social support (especially during childhood) seems to be the strongest protective factor for the development of PTSS according to our study. Consequently, parents and relatives must be involved in the psychosocial course of the illness from the very beginning. Additionally, social support is one of the most important factors of the development of SOC. A high SOC has been shown to be of protective character of PTSS in this study. Besides the positive influence on the development of PTSS, a low SOC has been associated with an increased risk of all-cause mortality in the general adult population [41]. This is especially alarming in the context of patients with CHD who, besides all medical advances and progress in cardiac and surgical care, remain chronically ill [1]. Furthermore, a strong SOC has been found to be a good predictor for successful changes in lifestyle in patients at risk of diabetes type 2. Consequently, SOC can be used as a target construct to improve patients’ mental and physical outcome and potentially even decrease mortality.

Even though the present study provides insights into factors associated with potential of developing PTSS in ACHD, the results should be interpreted with caution.

First, self-assessed measures do not provide a clinical diagnosis of PTSD. Consequently, we cannot refer to the presence of a diagnosed PTSD but to related symptoms (PTSS) being reported. Second, all findings are at risk for response bias as, except for medical parameters, all variables were self-reported. However, by assuring strict pseudonymization, utmost care was taken to minimize the potential for this bias. Third, the response rate was rather low and patients with severe mental health problems and large degrees of distress might be more prone to participate in this study which could lead to self-selection bias and affect external validity. This study was performed in a tertiary care center for adults with CHD. Therefore, the sample of patients does not represent the typical population with CHD seen by general practitioners or cardiologists. The prevalence of patients at risk in our institution is likely to be higher than that in regional hospitals or in regular cardiology departments. Therefore, the patient group might be biased toward patients with more symptomatic severity. Moreover, of the more than 360,000 people with ACHD living in Germany, only a small proportion receive specialized healthcare. It is completely unknown to what extent the collected data can be applied to this group of patients. Fourth, recall bias might occur as many questions were of retrospective nature. However, traumatic events are so affecting for an individual that recall bias does most likely not occur [42]. Fifth, due to its cross-sectional nature, it is not possible to draw any conclusions about directs or effects. Hence, longitudinal studies are needed to understand causality. Qualitative research is needed for a better understanding of (traumatic) experiences in ACHD and their resulting mental distress.

## Conclusion

Due to various reasons, ACHD are at risk of developing PTSS. Resilience, SOC, and social support seem to be of protective nature for the development of PTSS. Screening for resilience, SOC, and social support might offer a cost-effective and easy to use option for health promotion to detect patients at risk. Based on the findings of this study on the protective influence of social support, raising awareness, involving parents and relatives, seems to be highly important. Offering multidisciplinary care and education for patients for increasing resilience and SOC might provide a way of preventing the negative impact of mental distress, enhancing mental and consequently psychosocial outcomes, quality of life, and adherence in ACHD.

## Data Availability

Not applicable.
